# Supplementary material for: Neural signatures associated with temporal compression in the verbal retelling of past events
Source: Commun Biol. 2022 May 23;5:489. doi: 10.1038/s42003-022-03418-5 (PMC9126919; doi:10.1038/s42003-022-03418-5)
Supplement: Supplementary file 2 — Supplementary Information (new) [file 42003_2022_3418_MOESM2_ESM.pdf]

Supplementary Information

	All utterances	Precise	Summary
Number of utterances	203.1 (110.1), 114-468	108.1 (75.6), 36-280	54.3 (15.7), 34-91
Number of words per utterance	13.87 (2.39), 10.2-19.5	13.57 (2.1), 10.1-18.0	15.75 (3.34), 10.8-22.1
Duration per utterance (seconds)	6.83 (1.6), 4.4-10.0	6.29 (1.31), 4.1-8.7	8.21 (2.2), 4.9-12.2
Proportion of all utterances		0.47 (0.1), 0.3-0.71	0.3 (0.1), 0.16-0.40

Supplementary Table 1. Descriptive statistics of recall utterances
Entries list the group average value, the standard deviation in parentheses, and the minimum to maximum range of values across subjects.

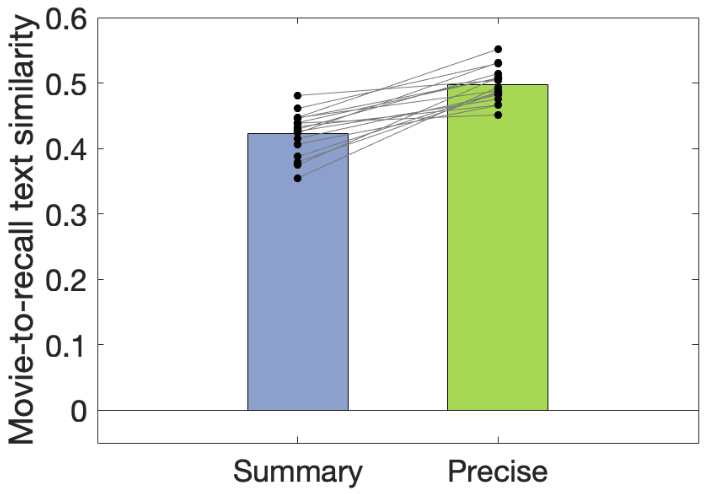

Supplementary Figure 1. Cosine similarity between text embeddings derived from movie annotations versus spoken recall, separated by recall type for each subject. Movie-to-recall text cosine similarity was separately computed for summary versus precise recall utterances. Each dot shows similarity values for an individual subject with lines connecting each subject's values for each recall type.

Region of interest	Schaefer parcel ID	Notes
Posterior medial cortex (PMC)	154-160; 363-367	All parcels labeled "PCC" in "DefaultA" network

Supplementary Table 2. Description of posterior medial cortex (PMC) ROI. List of Schaefer et al. (2017) 400 parcels used to create the posterior medial cortex (PMC) region of interest.

Supplementary Note 1. Testing for within-subject reinstatement effects in anterior hippocampus and posterior hippocampus

In addition to the parcel-level results reported in the main paper, we also tested for effects of 1) within-subject memory reactivation and 2) between-subject memory transformation in two anatomically-defined hippocampal ROIs: posterior hippocampus and anterior hippocampus.

For the within-subjects reactivation analysis at the micro-segment level, neither hippocampal ROI showed reliable reactivation effects during either summarized or temporally precise recall. In the posterior hippocampus, there was no reliable reactivation for micro-segments that are later recalled in a temporally precise manner, $t(16)= 1.5$, $p= 0.15$, for micro-segments that are later summarized, $t(16)= 1.10$, $p= 0.28$. This was also the case for anterior hippocampus: there was no reliable reactivation for micro-segments with temporally precise recall, $t(16)= 1.6$, $p= .13$, or for summarized micro-segments, $t(16)= 0.48$, $p= .42$.

We also tested for memory transformation effects in each hippocampal ROI, by computing encoding-to-recall similarity and recall-to-recall similarity across people at the 50-scene level, as described on pg. 25-26. In posterior hippocampus, neither the recall-to-recall (RR) nor movie-to-recall (MR) effect was significant (RR: $t(16)= -0.7$, $p= .52$, mean similarity: r=-0.004; MR: $t(16)= 0.66$, $p= .52$, mean similarity: r= 0.01). This was also the case in anterior hippocampus (RR: $t(16)= 0.67$, $p= .51$, mean similarity: r=0.007; MR: $t(16)= 0.36$, $p= .73$, mean similarity: r= 0.004). In addition, we found that recall-to-recall (RR) similarity did not exceed movie-to-recall similarity (MR) in either ROI (anterior hippocampus: RR minus MR similarity: r = -0.05; posterior hippocampus: RR minus MR similarity: r = -0.01).

Supplementary Note 2. List of stop words excluded from word counts of transcribed recall speech.

The following terms were considered stop words and thus were omitted from count of the number of words for utterances that contained them: a, an, and, are, as, at, be, by, for, from, has, in, is, its, of, on, that, the, to, was, were, will, with, uh, um, uhm, umm, uhh

Supplementary Note 3. Proportion of parcels from each functional network in the memory transformation mask

The proportion of tested parcels (i.e., those in the memory transformation mask in Figure 3a of the main text) where memory transformation scaled with summarization varied across networks: 44% of tested parcels in the Ventral Attention Network, 38% of the Fronto-Temporal parcels; 31% of the Visual Network Parcels; 28% of the DMN parcels; 22% of the Somato-Motor Parcels, and 20% of the Dorsal Attention Parcels.

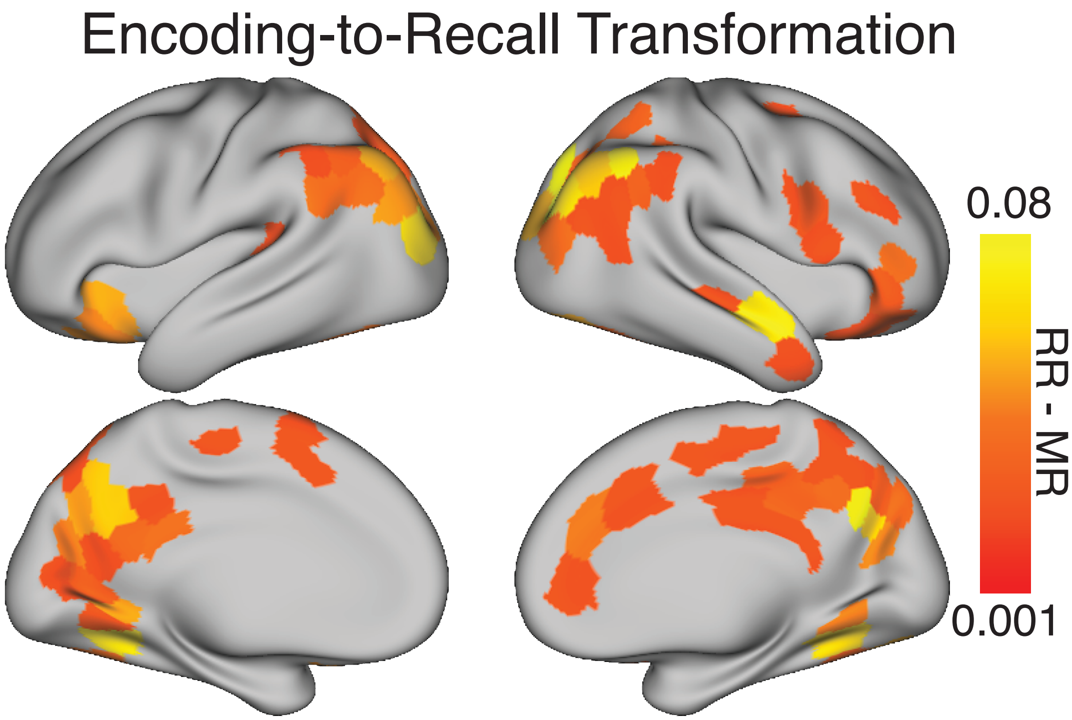

Supplementary Figure 2. Average memory transformation values (recall-to-recall minus movie-to-recall) from the searchlight analysis reported in Chen et al. 2017. Effects are shown in parcels where either recall-to-recall or movie-to-recall (between subjects) was reliable. For each reliable parcel (shown in Figure 3a, colored and light gray parcels), the memory transformation values observed in the parcel's searchlight centers were averaged together.

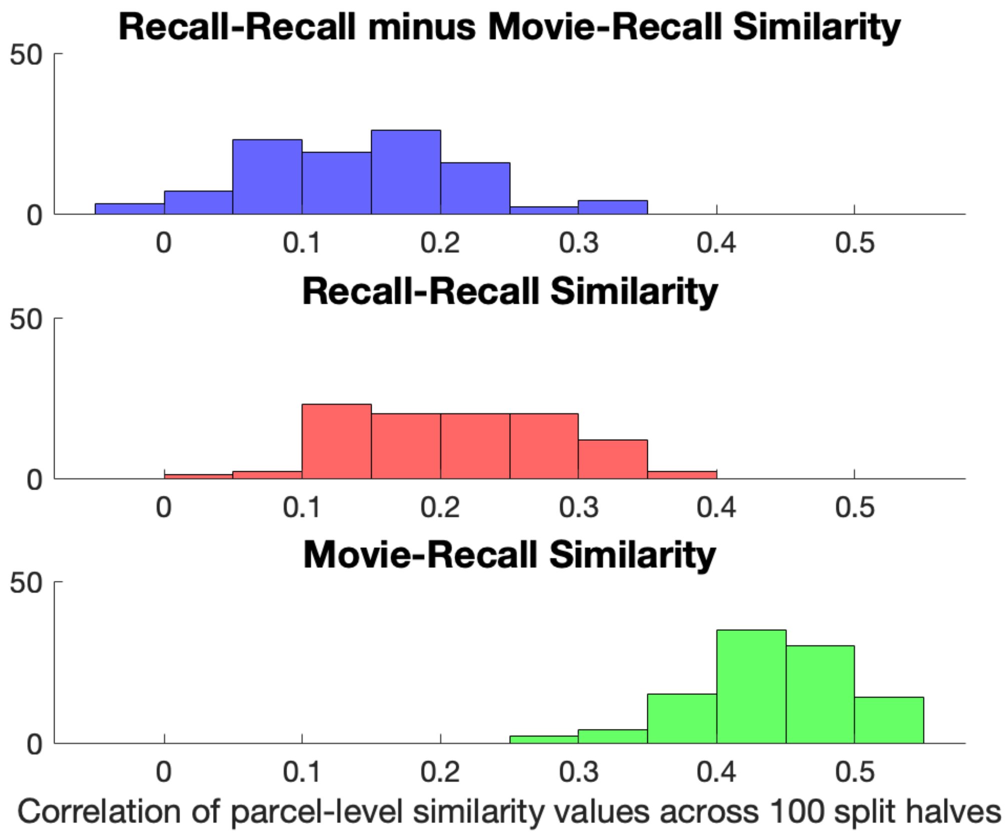

Supplementary Figure 3. Measures of split-half test-retest reliability for between-subjects pattern similarity across parcels. There is no previous work to evaluate the test-retest reliability of our fMRI measurement of *memory transformation*, i.e., where *recall-recall* correlations (similarity of brain activity patterns across subjects during *recall*) exceed *movie-recall* correlations (similarity of *movie* brain activity patterns with *recall* brain activity patterns across subjects). To compute test-retest reliability, we split the data in half (two sub-groups with random assignment, n=9 and n=8) and computed 1) *movie-recall* similarity 2) *recall-recall* similarity and 3) the difference between 1 and 2 (i.e., *memory transformation*) in each parcel. For more details on how these measures are computed, see pg. 26. We excluded parcels that did not show reliable effects for either 1) or 2) in the whole-group data, limiting analyses to the colored and light gray parcels depicted in Figure 3a (174/400 parcels). Thus, for each of the two sub-groups, we computed the three measures in each of the 174 parcels. We then computed the Pearson correlation (r-value) between the 174 memory transformation values across the two sub-groups, which quantifies the extent to which parcel-level memory transformation values are similar across the two sub-groups. We repeated this analysis 100 times with random splits of the 17 subjects into two sub-groups. The histogram below (top row) shows the distribution of r-values (correlation of 174 memory transformation values across the two random sub-groups). The distribution average was r=0.14. Reliability (the distribution average) for recall-to-recall (measure #1, middle row) was r=.21 and reliability for movie-to-recall was r=.44 (measure #2, bottom row). In sum, for all three measures, we found consistently positive correlations between the parcel values for random split-halves of the data. Note that in order to avoid performing inter-subject pattern similarity analyses with too little data, scenes were excluded if they were

recalled by less than five participants in each split-half group, leading to the inclusion of an average of 13.5/50 scenes across each the 100 random samples.

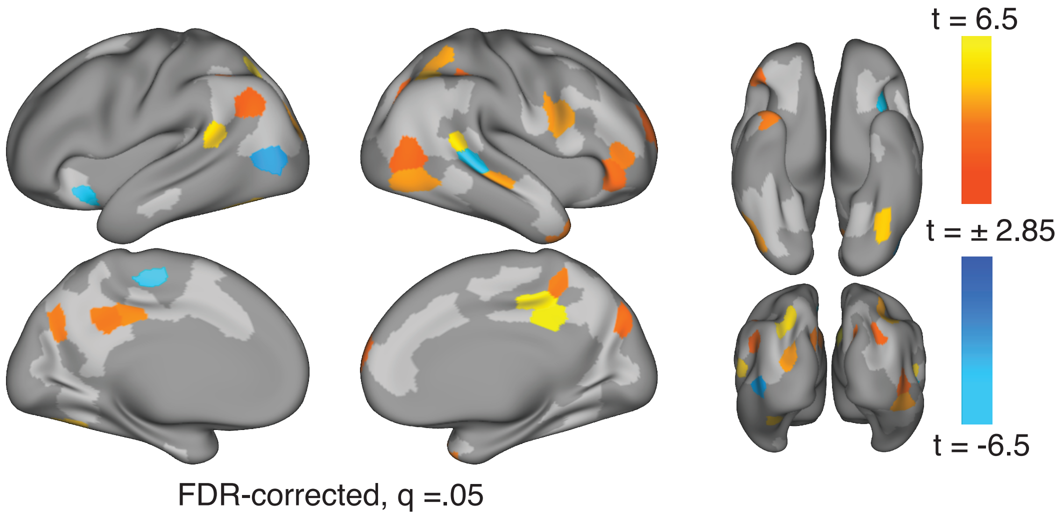

Supplementary Figure 4. Parcel-level map of the relationship between memory transformation and scene-level temporal compression factor (TCF) scores. This relationship was only tested in parcels that showed reliable memory transformation effects (shown as light gray and colored parcels, see Figure 3a of main text). The parcel values yielded by this analysis are moderately correlated (r=.65) with the parcel values reported in the main manuscript text (see Figure 4b).
