# Supplementary material for: Neural signatures associated with temporal compression in the verbal retelling of past events
Source: Commun Biol. 2022 May 23;5:489. doi: 10.1038/s42003-022-03418-5 (PMC9126919; doi:10.1038/s42003-022-03418-5)
Supplement: Supplementary file 3 — Description of Additional Supplementary Files [file 42003_2022_3418_MOESM3_ESM.pdf]

Description of Additional Supplementary Files

File name: Supplementary Data 1
Description: Data tables to reproduce figures 1b-c, 2a, 4a, 4c.
