# Supplementary material for: Neural signatures associated with temporal compression in the verbal retelling of past events
Source: Commun Biol. 2022 May 23;5:489. doi: 10.1038/s42003-022-03418-5 (PMC9126919; doi:10.1038/s42003-022-03418-5)
Supplement: Supplementary file 5 — Reporting Summary [file 42003_2022_3418_MOESM5_ESM.pdf]

nature portfolio

Corresponding author(s): Musz, Elizabeth

Last updated by author(s): Apr 25, 2022

Reporting Summary

Nature Portfolio wishes to improve the reproducibility of the work that we publish. This form provides structure for consistency and transparency in reporting. For further information on Nature Portfolio policies, see our Editorial Policies and the Editorial Policy Checklist.

Statistics

For all statistical analyses, confirm that the following items are present in the figure legend, table legend, main text, or Methods section.

n/a	Confirmed	
☐	☒	The exact sample size (*n*) for each experimental group/condition, given as a discrete number and unit of measurement
☐	☒	A statement on whether measurements were taken from distinct samples or whether the same sample was measured repeatedly
☐	☒	The statistical test(s) used AND whether they are one- or two-sided<br>*Only common tests should be described solely by name; describe more complex techniques in the Methods section.*
☐	☒	A description of all covariates tested
☐	☒	A description of any assumptions or corrections, such as tests of normality and adjustment for multiple comparisons
☐	☒	A full description of the statistical parameters including central tendency (e.g. means) or other basic estimates (e.g. regression coefficient) AND variation (e.g. standard deviation) or associated estimates of uncertainty (e.g. confidence intervals)
☐	☒	For null hypothesis testing, the test statistic (e.g. *F*, *t*, *r*) with confidence intervals, effect sizes, degrees of freedom and *P* value noted<br>*Give P values as exact values whenever suitable.*
☒	☐	For Bayesian analysis, information on the choice of priors and Markov chain Monte Carlo settings
☒	☐	For hierarchical and complex designs, identification of the appropriate level for tests and full reporting of outcomes
☐	☒	Estimates of effect sizes (e.g. Cohen's *d*, Pearson's *r*), indicating how they were calculated

Our web collection on statistics for biologists contains articles on many of the points above.

Software and code

Policy information about availability of computer code

Data collection	The Psychophysics Toolbox (http://psychtoolbox.org/) for MATLAB was used to display the movie and to synchronize stimulus onset with MRI data acquisition. Participants' speech was recorded using a customized MR-compatible recording system (FOMRI II; Optoacoustics Ltd.).
Data analysis	In house MATLAB 2018 scripts were used to run the analyses.

For manuscripts utilizing custom algorithms or software that are central to the research but not yet described in published literature, software must be made available to editors and reviewers. We strongly encourage code deposition in a community repository (e.g. GitHub). See the Nature Portfolio guidelines for submitting code & software for further information.

Data

Policy information about availability of data

All manuscripts must include a data availability statement. This statement should provide the following information, where applicable:

- Accession codes, unique identifiers, or web links for publicly available datasets
- A description of any restrictions on data availability
- For clinical datasets or third party data, please ensure that the statement adheres to our policy

The fMRI data that support the findings of this study are available online at http://dataspace.princeton.edu/jspui/handle/88435/dsp01nz8062179. The behavioral data that support the findings of this study, as well as the parcel-level memory transformation values, are available online at https://osf.io/8gmes/

Field-specific reporting

Please select the one below that is the best fit for your research. If you are not sure, read the appropriate sections before making your selection.

☐ Life sciences ☒ Behavioural & social sciences ☐ Ecological, evolutionary & environmental sciences

For a reference copy of the document with all sections, see nature.com/documents/nr-reporting-summary-flat.pdf

Behavioural & social sciences study design

All studies must disclose on these points even when the disclosure is negative.

Study description	quantitative experimental
Research sample	Sample is 17 participants, age 18-26, recruited from the Princeton community. All participants were right-handed native English speakers. The data was initially collected and described in Chen, J., Leong, Y., Honey, C. et al. Shared memories reveal shared structure in neural activity across individuals. Nat Neurosci 20, 115–125 (2017). https://doi.org/10.1038/nn.4450
Sampling strategy	Participants were randomly sampled from those who met the age, language, and handedness criteria. In addition, all participants had not watched any episodes of Sherlock before the experiment. No statistical methods were used to predetermine sample sizes, but our sample sizes are similar to those reported in previous publications.
Data collection	Participants' neural data was collected on a 3T Siemans Skyra scanner and participants' verbal recall was collected with an MRI-compatible microphone and then transcribed. The movie was projected using an LCD projector onto a rear-projection screen located in the magnet bore and viewed with an angled mirror and audio was delivered via in-ear headphones. Only the researcher and participants were present during data collection. There was only one experimental condition, and esearchers were not blind to the hypothesis or experimental condition during testing. For more details, see Chen, J., Leong, Y., Honey, C. et al. Shared memories reveal shared structure in neural activity across individuals. Nat Neurosci 20, 115–125 (2017). https://doi.org/10.1038/nn.4450
Timing	Data were collected between June and December 2013
Data exclusions	Data from 5 of the original 22 participants were discarded due to excessive head motion (greater than 1 voxel; 2 participants), because recall was shorter than 10 min (2 participants), or for falling asleep during the movie (1 participant). These exclusion criteria were pre-established.
Non-participation	No participants dropped out/declined participation.
Randomization	Participants were not allocated into experimental groups.

Reporting for specific materials, systems and methods

We require information from authors about some types of materials, experimental systems and methods used in many studies. Here, indicate whether each material, system or method listed is relevant to your study. If you are not sure if a list item applies to your research, read the appropriate section before selecting a response.

Materials & experimental systems

n/a	Involved in the study	
☒	☐	Antibodies
☒	☐	Eukaryotic cell lines
☒	☐	Palaeontology and archaeology
☒	☐	Animals and other organisms
☐	☒	Human research participants
☒	☐	Clinical data
☒	☐	Dual use research of concern

Methods

n/a	Involved in the study	
☒	☐	ChIP-seq
☒	☐	Flow cytometry
☐	☒	MRI-based neuroimaging

Human research participants

Policy information about studies involving human research participants

Population characteristics	See above
Recruitment	Participants were recruited using flyers posted on Princeton University campus and an announcement posted to a university-affiliated participant recruitment website

Ethics oversight	Experimental procedures approved by the Princeton University Institutional Review Board.

Note that full information on the approval of the study protocol must also be provided in the manuscript.

Magnetic resonance imaging

Experimental design

Design type	Event-related design
Design specifications	Participants completed three scanning runs. The first two were for movie viewing, which were 23 and 25 minutes long. The duration of the third scanning run, for collecting free recall data, varied in duration according to how long the participant spoke but lasted at least 10 minutes. Data were later segmented into discrete events according to plot/ character changes, as determined by an independent annotator who was blind to the experimental hypotheses.
Behavioral performance measures	Verbal responses were recorded via microphone and transcribed. Transcripts were written of the audio recording of each participant's spoken recall. Timestamps were then identified that separated each audio recording into the same 50 scenes that had been previously selected for the audiovisual stimulus. A scene was counted as "recalled" if the participant described any part of the scene. Scenes were counted as "out of order" if they were initially skipped and then described later.

Acquisition

Imaging type(s)	functional
Field strength	3 Telsa
Sequence & imaging parameters	Data were collected with a 20-channel head coil. Functional images were acquired using a T2*-weighted echo-planar imaging (EPI) pulse sequence (TR 1,500 ms, TE 28 ms, flip angle 64, whole-brain coverage 27 slices of 4 mm thickness, in-plane resolution 3 × 3 mm2, FOV 192 × 192 mm2), with ascending interleaved acquisition. Anatomical images were acquired using a T1-weighted MPRAGE pulse sequence (0.89 mm3 resolution).
Area of acquisition	whole brain

Diffusion MRI ☐ Used ☒ Not used

Preprocessing

Preprocessing software	Preprocessing was performed in FSL (http://fsl.fmrib.ox.ac.uk/fsl), including slice time correction, motion correction, linear detrending, high-pass filtering (140 s cutoff).
Normalization	The functional volumes were co-registered and were affline-transformed to a template brain (MNI). Functional images were resampled to 3 mm isotropic voxels for all analyses.
Normalization template	images were registered to the MNI152 template
Noise and artifact removal	Motion was minimized by instructing participants to remain very still while speaking and by stabilizing participants' heads with foam padding. Artifacts generated by speech may introduce some noise, but they cannot induce positive results, as our analyses depend on spatial correlations between sessions (movie–recall or recall–recall). For more details, cf. Chen et al. (2017)
Volume censoring	The first two volumes during scanning were omitted

Statistical modeling & inference

Model type and settings	Inter-subject and within-subject spatial pattern correlation (spatial ISC) were performed by first computing event-level multi-voxel patterns during movie viewing and verbal recall in each participant. To compute spatial ISC, each participants' event-level spatial patterns were correlated with the group-average pattern from the other 16 participants. For the reinstatement analysis, repeated measures (paired t-tests) were used to compare each participant's movie-recall event-level pattern similarity for events that they later summarized versus precisely recalled. These tests were performed in each of 400 parcels, defined an independent whole-brain resting-state parcellation (Schaefer et al., 2018).<br>For the memory transformation analysis, inter-subject spatial correlations were computed for both the movie-viewing and verbal recall data. In each case, statistical significance was determined by shuffling scene labels to generate a null distribution. For linking memory transformation to summarization, each subject's individual summarization scores were correlated with the group-level memory transformation values (limited to 174/400 parcels that showed either reliable movie-recall or recall-recall inter-subject spatial correlations). In a random effects analysis, the resulting transformation-summarization correlation values were submitted to a one-sample t-test versus zero (two-tailed).
Effect(s) tested	Inter-subject spatial pattern correlation (spatial ISC) for movie scenes during movie viewing versus verbal recall across parcels (memory transformation analysis); random effects analysis to test for relationship between participant-level summarization scores and group-level memory transformation values; spatial ISC during verbal recall versus movie viewing; within-subjects,

scene-level movie-recall pattern across parcels (reinstatement analysis) for scenes that are summarized versus precisely recalled

Specify type of analysis: ☐ Whole brain ☒ ROI-based ☐ Both

Anatomical location(s)	All reported similarity and correlation values were computed in 400 parcels from an independent whole-brain resting-state parcellation (Schaefer et al. Local-global parcellation of the human cerebral cortex from intrinsic functional connectivity MRI. Cerebral cortex. 2018 Sep 1;28(9):3095-114). Each parcel is associated with one of seven functional networks (see Figure 3B).
Statistic type for inference (See Eklund et al. 2016)	Voxel-wise statistical tests (e.g., spatial ISC) were performed in each parcel.
Correction	Following all statistical tests, we corrected for multiple comparisons across parcels by controlling the False Discovery Rate (FDR) (Benjamini and Hochberg, 1995) using q criterion = 0.05 (and in one case at q = 0.10, as stated in the manuscript).

Models & analysis

n/a	Involved in the study
☒	☐ Functional and/or effective connectivity
☒	☐ Graph analysis
☒	☐ Multivariate modeling or predictive analysis
